# Genes Encoding Toxin of *Clostridium difficile* in Children with and without Diarrhea

**DOI:** 10.1155/2014/594014

**Published:** 2014-04-29

**Authors:** Victor R. C. Merino, Viviane Nakano, Sydney M. Finegold, Mario J. Avila-Campos

**Affiliations:** ^1^Anaerobe Laboratory, Department of Microbiology, Institute of Biomedical Science, University of São Paulo, São Paulo, SP, Brazil; ^2^Veterans Affairs, West Los Angeles Medical Center, Los Angeles, CA, USA; ^3^Department of Medicine, School of Medicine, University of California, Los Angeles, CA, USA; ^4^Department of Microbiology, Immunology and Molecular Genetics, School of Medicine, University of California, Los Angeles, CA, USA

## Abstract

The presence of gene 16S rRNA and genes encoding toxin A (*tcdA*), toxin B (*tcdB*), and binary toxin (*cdtA/cdtB*) of *Clostridium difficile* in stool samples from children with (110) and without (150) diarrhea was determined by using a TaqMan system. Fifty-seven (21.9%) out of 260 stool samples harbored the 16S rRNA gene. The genetic profile of *tcdA+/tcdB−* and *cdtA+/cdtB+* was verified in one *C. difficile*-positive diarrhea sample and of *tcdA+/tcdB+* in three *C. difficile*-positive nondiarrhea samples. The presence of *tcdA+/tcdB+* in stools obtained from children without diarrhea, suggests that they were asymptomatic carriers of toxigenic strains.

## 1. Introduction

*Clostridium difficile* is the primary etiological agent of antibiotic-associated diarrhea and pseudomembranous colitis and is recognized as a cause of nosocomial diarrhea worldwide [1]. The pathogenic effects of* C. difficile *are mucosal damage to the colon that is caused by the production of toxin A (308 kDa) and/or toxin B (270 kDa) [2].

Some* C. difficile* strains also produce a binary toxin encoded by* cdtA* and* cdtB *genes, and both genes have been observed in strains producing* C. difficile*-associated diarrhea (CDAD) [3]. In Brazil, few studies have shown the presence of toxigenic* C. difficile *harboring* tcdA *and/or* tcdB* but not the binary toxin genes in stool samples from children with acute diarrhea [4, 5].

The cytotoxic assays performed from stool samples or isolated strains for detecting toxigenic* C. difficile *are considered the “gold standard” [6]. Other methods such as culture and enzyme immunoassay (EIA) are not specific for the identification of these toxigenic strains, but EIA method is used in most laboratories worldwide [7].

In this study, a quantitative detection of genes encoding toxins A and B and binary toxin directly from* C. difficile*-positive stool samples of children with and without acute diarrhea was determined.

## 2. Methods

### 2.1. Sample Collection and DNA Extraction

Two hundred sixty stool samples were obtained from 110 children with diarrhea and from 150 children without diarrhea, aged from 1 month to 8 years old. Stool samples were collected from March 2008 through November 2010. Children did not display other illnesses or comorbidities than diarrhea, and they were not under antibiotic treatment for at least three months prior to the sample collection. Diarrhea was defined as three or more unformed stools in the 24 h prior to enrollment. The Ethics Committee of the Biomedical Science Institute at the University of Sao Paulo (number 743/CEP) approved this study. Total bacterial genomic DNA was obtained from stools by using a QIAamp DNA stool minikit (Qiagen, Germany) according to the manufacturer's instructions.

All toxigenic* C. difficile*-positive stool samples were evaluated for the presence of the toxins A/B with an enzyme immunoassay (EIA) by using a commercial kit Xpect* C. difficile* Toxin A/B Test (Remel, USA).

### 2.2. PCR Amplification

Real-time PCR assays were carried out in duplicate and performed in a total volume of 25 *μ*L, containing 2X TaqMan Universal Master Mix (Applied Biosystems, USA), 100 *μ*M of each primer, 100 *μ*M of TaqMan probe, and 2 ng of DNA. Amplifications were performed in a thermal cycler programmed as follows: denaturation at 95°C for 10 min, followed by 45 cycles of two steps: denaturation at 95°C for 15 s and an annealing temperature at 60°C for 1 min, for detecting* C. difficile* 16S rRNA gene; and annealing temperatures for different toxin-encoding genes were as follows: at 51°C for detecting* tcdA* and* cdtA* genes and at 58°C for detecting* tcdB* and* cdtB* genes. The primer/probe sets used are shown in Table 1.

Bacterial DNA amplifications were adjusted using *R* ^2^ values > 0.900. A sample was considered positive for a target gene when the detected fluorescence generates a curve above the background fluorescence, which was established by Rotor Gene 6000 analytical software (Corbett Life Science, Australia).

### 2.3. Statistical Analysis

Mean values ± SD were calculated for each toxin gene using a Student's *t*-test. A difference of *P* < 0.05 was considered statistically significant (GraphPad Prism Software Inc.).

## 3. Results

By using a commercial kit Xpect, no toxin-positive stool sample was observed. Fifty-seven (21.9%) out of 260 stool samples analyzed harbored* C. difficile *(20 from children with diarrhea and 37 from children without diarrhea). Twenty* C. difficile*-positive diarrhea samples were obtained from children between six months and 7 years old, and, among them, a 1-year-old child harbored the* tcdA* gene and another 1-year-old child (*tcdA−/tcdB−*) harbored both binary toxin genes (*cdtA* and* cdtB*). Also, the 37 (24.6%)* C. difficile*-positive nondiarrhea samples were obtained from 1-to-5-year old children, and, of them, three children (a 1 year old and 2 years old) harbored both genes* tcdA* and* tcdB* (Table 2). None of the* C. difficile*-positive nondiarrhea samples harbored the binary toxin genes.

The presence of the target genes and their respective copy numbers are shown in Table 2. The* tcdA* was observed in diarrhea sample with a value of log⁡_10_⁡2.1. Similarly, binary toxin genes were observed in log⁡_10_⁡2.7 (*cdtA*) and log⁡_10_⁡3.7 (*cdtB*). Moreover, in nondiarrhea samples* tcdA* was detected from log⁡_10_⁡1.3 to log⁡_10_⁡3.6 and* tcdB* from log⁡_10_⁡3.2 to log⁡_10_⁡5.3. The specific primer/probe sets here used showed an accurate standard curve over a 5-log-unit linear range that permitted the copy number determination from standard amplifications and detection to a limit of the 100 copies of the target DNA in unknown samples. In this study,* C. difficile* was detected in diarrhea (18.2%) and nondiarrhea (24.6%) stool samples.

## 4. Discussion

In Brazil, there are few epidemiological studies involving this pathogen as a cause of diarrhea in children [4, 8]. Since a stool sample harbored the profile of toxigenicity* tcdA+/tcdB−* and another* cdtA+/cdtB+*, it suggested a low circulation of toxigenic* C. difficile* in Brazilian children, and the acute diarrhea in children could have been caused by other enteropathogens than* C. difficile*, like typical viral or bacterial pathogens, such as* Norovirus*,* Rotavirus*,* Salmonella*, or* Campylobacter*, and this needs to be investigated.

The presence of toxins A/B and binary toxin-producing* C. difficile* in diarrhea outbreak has been associated to severe CDAD processes [9]. On the other hand, it is possible that strains can harbor toxins A/B and/or binary toxin genes without any expression, and it could explain the failure to detect the presence of toxins when the EIA was used.

The quantitative PCR by using the TaqMan system provided a direct detection of* tcdA/tcdB* and* cdtA/cdtB* from diarrhea and nondiarrhea stool samples, and it could determine a rapid bacterial screening in patients with acute diarrhea, and, due to the high sensitivity to detecting* C. difficile* strains, this assay has been useful in surveillance of nosocomial diarrhea when required [10, 11].

The presence of toxin genes (*tcdA+/tcdB+*) in stool samples obtained from three children without diarrhea suggests that they were asymptomatic carriers of toxigenic strains. In addition, an asymptomatic colonization by toxigenic* C. difficile* was recently described in neonates, suggesting that healthy children display some tolerance to toxigenic strains, and it could play a role in the development of the intestinal immune system [12–15].

Our results suggest that more studies are necessary to determine the real role of toxigenic* C. difficile* strains and their asymptomatic colonization in the human intestinal tract, mainly in children.

## Figures and Tables

**Table 1:** Genes and primer/probe sets used to detect *C. difficile* and genes encoding toxins.

Genes	Oligonucleotides 5′→3′	Melting temperature (°C)	References
16S rRNA*	F-TTG AGC GAT TTA CTT CGG TAA AGA R-TGT ACT GGC TCA CCT TTG ATA TTC A P-CCA CGC GTT ACT CAC CCG TCC G	60	[16]

tcdA**	F-CAG GGC TAA TAG TTT GTT TAC AGA ACA R-CAA CAT CTA AAT ATA CTC CGC CAA AA P-TTA TAG TCA GCA GCT AGG ATT TCC ACG ATT TAA CAA CTC C	51	[17]

tcdB**	F-AGC AGT TGA ATA TAG TGG TTT AGT TAG AGT TG R-CAT GCT TTT TTA GTT TCT GGA TTG AA P-CAT CCA GTC TCA ATT GTA TAT GTT TCT CCA	58	[17]

cdtA**	F-GAT CTG GTC CTC AAG AAT TTG GTT R-GCT TGT CCT TCC CAT TTT CGA TT P-AAC TCT TAC TTC CCC TGA AT	51	[17]

cdtB**	F-AAA AGCT TCA GGT TCT TTT GAC AAG R-TGA TCA GTA GAG GCA TGT TCA TTT G P-CAA GAG ATC CGT TAG TTG CAG CAT ATC CAA TTG T	58	[17]

*Used to detect *C. difficile*.

**Used to detect toxigenic *C. difficile*.

**Table 2:** Quantitative detection of *Clostridium difficile* and target genes in diarrhea and nondiarrhea samples.

Target gene	Diarrhea (*n* = 110)		Nondiarrhea (*n* = 150)		*P*
	Number (range of log_10_⁡ )	Mean ± SD	Number (range of log_10_⁡ )	Mean ± SD	
16S rRNA^∗^	20 (1.2–6.9)	4.0 ± 1.7	37 (1.3–8.0)	4.8 ± 1.5	0.063
*tcdA *	1 (2.1)	ND	3 (1.3–7.6)	2.9 ± 1.3	ND
*tcdB *	0 (0)	ND	3 (3.2–5.3)	4.6 ± 1.2	ND
*cdtA *	1 (2.7)	ND	0 (0)	ND	ND
*cdtB *	1 (3.7)	ND	0 (0)	ND	ND

*Used to detect *C. difficile*.

ND: not determined.
